# Editorial: World diabetes day 2024: exploring mechanisms, innovations, and holistic approaches in diabetes care

**DOI:** 10.3389/fendo.2026.1808396

**Published:** 2026-03-06

**Authors:** Åke Sjöholm, Ajay D. Rao

**Affiliations:** 1 Department of Internal Medicine, Division of Endocrinology and Diabetology, Gävle Hospital and University of Gävle, Gävle, Sweden; 2 Center for Metabolic Disease Research, Lewis Katz School of Medicine, Temple University, Philadelphia, PA, United States

**Keywords:** depression, GLP-1, islet, prediabetes, SGLT2

Diabetes continues to pose a significant global health challenge, affecting millions and driving complications such as cardiovascular disease, neuropathy, nephropathy, retinopathy, and hepatic dysfunction. This Research Topic attempts to harness the momentum of World Diabetes Day 2024 by bringing together leading research on the underlying mechanisms, emerging therapies, and interdisciplinary approaches to managing and preventing diabetes and its complications.

This Research Topic encompasses efforts to integrate molecular, clinical, and psychosocial perspectives to explore innovative strategies and actionable insights for advancing diabetes care. By addressing both established and evolving research areas, we hope to foster collaborations and translate findings into improved outcomes for patients across diverse populations.

There are 25 research articles in the Research Topic (Frontiers | World Diabetes Day 2024: Exploring Mechanisms, Innovations, and Holistic Approaches in Diabetes Care). Below we highlight some of these and their main findings and conclusions.

There is a rapid and alarming increase in overweight and obesity worldwide, which also brings with it a corresponding increase in the dysmetabolic syndrome, in which insulin resistance is an important pathogenetic factor. Impaired glucose tolerance is a central component of this syndrome. Before manifest diabetes occurs, these prediabetic stages have usually been ongoing for many years. However, vascular complications of this prediabetic hyperglycemia and other consequences of insulin resistance (*e.g.* dyslipidemia) develop throughout this prediabetic period. This is the explanation why almost half of all patients with newly diagnosed type 2 diabetes (T2D) already have some form of late complication (*e.g.* sensory neuropathy or background retinopathy) at the time of diagnosis. In many cases, prediabetes or previously unknown diabetes is only discovered in connection with myocardial infarction and other life-threatening conditions (1). It follows that it is important to observe prediabetes early and to be vigilant about it in risk individuals (*e.g.* age > 45 years, abdominal obesity, physical inactivity, hypertension, sleep apnea syndrome, heredity for diabetes or premature cardio- or cerebrovascular disease).

In addition to this increased risk, the condition carries a high risk over time of progression from prediabetes to manifest T2D (2–5). If no intervention is carried out, the natural course is that a significant proportion will progress to manifest diabetes within 5–10 years. The proportion to which this occurs has been reported to vary greatly, depending, among other things, on ethnicity and how close the glucose levels are to the diabetes threshold. It is often stated that the risk is increased 3–10 times compared with normoglycemic individuals (1). The Diabetes Prevention Program (DPP) study showed that 35% of patients with impaired glucose tolerance (IGT) progressed to overt diabetes over a four-year period (6).

The paper by Feng et al. addresses prediabetes and its relation to different surrogate measures of insulin resistance. More specifically, they studied the link between triglyceride-glucose (TyG) indices and prediabetes in a Chinese population. In a retrospective cohort study with > 160,000 Chinese adults free of diabetes at baseline they report that both TyG and TyG-BMI showed non-linear associations with incident prediabetes over a three year period. Women and adults younger than 45 years showed conspicuously steeper risk increases. These findings may be harnessed to advantage in identifying high-risk insulin resistance states portending dysglycemia.

Guo et al. also employed the TyG index to evaluate the predictive utility of postprandial glucose levels for insulin resistance in subjects with T2D. While studies of glucose tolerance have traditionally relied on 2-hour glucose levels after a 75 gram oral glucose tolerance test (OGTT), recent reports have identified the 1-hour glucose level as a useful and informative complement (7). Study participants were subjected to a 125-gram standard steamed buns meal test, which is considered more physiologic than OGTT as both carbohydrates, fat and protein contents in the meal impact postprandial glucose. The authors found that the 1-hour glucose level shows a stronger association with the TyG index than the 2-hour glucose level. These findings lend support to the utility of the 1-hour glucose level as a sensitive marker for early identification of insulin resistance.

The paper by Li et al. investigated the utility of a machine learning model for predicting the risk of diabetic nephropathy in T2D patients. Diabetic kidney disease is one of the most common and serious late complications of T2D. It is estimated that 30-40% of patients with diabetes sooner or later will develop diabetic nephropathy to varying degrees. Similarly, T2D is the dominant cause of chronic kidney disease. While some diabetes complications, such as heart attack and stroke, have decreased over time, the opposite is seen with diabetic kidney disease. In patients with end-stage renal failure, diabetes carries an extremely increased risk of premature mortality with a 5-year prognosis on par with cancer. It is therefore paramount to find means of facilitating early detection and intervention. Authors found that the developed XGBoost machine learning model could optimally predict the occurrence of diabetic kidney disease in patients with T2D and they also report an online prediction calculator, offering an accessible and practical tool.

In the paper by Du et al., authors performed a meta-analysis to address whether antenatal depression in pregnant women is associated with an increased risk of gestational diabetes mellitus (GDM), an important area in which the literature reports conflicting results. By systematically interrogating several databases for observational studies, authors found that maternal depression during pregnancy was significantly associated with an increased risk (~ 37% relative increase) of GDM. Authors rightfully conclude that screening for depression in early pregnancy may represent a potential strategy to reduce the risk of GDM and improve maternal health outcomes.

Kempf et al. made a systematic review to compare the effects of large-sized formula diet-based lifestyle interventions *vs*. modern pharmacological antidiabetic treatments on body weight and glycated hemoglobin (HbA_1c_) reduction in obese T2D patients. By searching the PubMed database, authors found that formula diet-based lifestyle intervention might improve weight loss to a greater extent than antidiabetic drugs with comparable long-term glycemic control. These are important findings, but it remains to be seen whether the results can be extended longer than the < 12 months studied here. Additionally, while lifestyle intervention is the basis in T2D treatment this is usually eventually combined with pharmacologic treatment as T2D is a progressive disease with a slow but relentless loss of insulin production (8). Moreover, while weight loss through improved lifestyle of course is beneficial overall, it is somewhat disconcerting that it has proven difficult to show an impact on hard cardiovascular endpoints (9).

Gittens et al. conducted a retrospective pre-post secondary data analysis of Medicaid recipients with T2D to investigate the effects of health coaching on HbA_1c_, hospitalizations, and outpatient visits. They found that health coaching was associated with improved glycemic control and increased ambulatory care engagement. These encouraging findings show the importance and value of patient-centered interventions in a chronic disease like T2D, where lifestyle influence glycemic control to such a large extent. However, in clinical reality a major challenge is to get the patients who are in the greatest need to improve their lifestyle to accept participating in health coaching programs. From experience, it tends to be people who are already actively interested in and understand the importance of a healthy lifestyle that take part of health coaching programs, whereas others who need it most often abstain.

Wu et al. contributed an interesting review on the latest class of antidiabetic agents, sodium–glucose cotransporter 2 inhibitors (SGLT2i), with emphasis on their non-glycemic effects. SGLT2 in the renal tubule reabsorbs most of the glucose and sodium ions excreted in the primary urine, which in diabetes can be significant amounts. Inhibitors of SGLT2 thus reduce this reabsorption, which consequently leads to increased glucosuria and osmotic diuresis and thus better glycemic control, weight loss and slightly lower blood pressure (10, 11). This mechanism is thus independent of insulin. SGLT2i have shown impressive effects on mortality and cardiovascular and renal morbidity in randomized clinical trials in both diabetic and non-diabetic patients (12–14), suggesting that much of these salutary effects are not mediated by changes in glycemia. Conversely, it has also become increasingly evident that T2D is not just a disorder of blood glucose (although it is diagnosed that way), but a silent and systemic metabolic cardiorenal disease requiring a multifactorial approach that simultaneously addresses several risk factors (*e.g.* glycemia, lipids, blood pressure) to achieve maximal protection against cardiovascular disease and mortality (15, 16). Given the strong organ protective effects of SGLT2i noted in clinical trials, there is a great need for studies providing mechanistic insights into the underlying pathophysiology. SGLT2i possess many pleiotropic properties, besides promoting glucosuria, *e.g.* metabolic reprogramming, fuel switching, remodeling cardiac structure, and enhancing myocardial metabolism by switching to ketones as fuels. This review investigates the different roles of SGLT2i in metabolic regulation and cardiovascular protection.

The paper by Chen et al. deals with the highly interesting interface between hepatic function and glucose metabolism, more specifically non-alcoholic fatty liver disease (NAFLD) and prediabetes in the form of impaired fasting glucose. In a commendable effort, authors carried out a retrospective cohort study and analyzed health examination data collected from > 75,000 Chinese adults, leveraging the Hepatic Steatosis Index (HSI), a simple screening tool and surrogate marker for NAFLD. Authors conclude that elevated baseline HSI is a significant risk factor for developing impaired fasting glucose in Chinese adults and highlight the potential utility of HSI in identifying individuals at risk of dysglycemia.

Li and Wang performed a single-center, prospective observational cohort study to investigate the role of CD36 in diabetic kidney disease (DKD), with particular emphasis on its role in renal lipotoxicity. CD36 plays a key role in lipid accumulation and inflammation. The authors studied a cohort of T2D patients with early-stage DKD in which all patients were on metformin therapy. They aimed to evaluate the impact of 12 weeks of treatment with GLP-1RA or insulin on CD36 levels and their association with renal function in DKD patients. GLP-1RAs are widely used against T2D and one of these, semaglutide, has shown robust renoprotective effects (17). The main result was that GLP-1RAs significantly reduce CD36 and urinary albumin excretion in patients with early DKD, and was found to be superior to insulin in renal protection. Future mechanistic studies should address whether lowered CD36 indeed mediates the effects of GLP-1RA in preserving kidney function, but pending these CD36 may nonetheless serve as a biomarker for DKD and its progression.

In the paper by Aliyu et al., authors assessed the performance of several clinically relevant surrogate measures of insulin resistance (IR) and estimated the prevalence of IR in a large population-based cohort. This is important work as > 90% of T2D patients show IR. Studying > 7,800 fasting participants from the Qatar biobank, individuals were considered insulin sensitive if lean, not diagnosed with diabetes, no hypertriglyceridemia, and not on lipid-lowering drugs, while individuals with T2D were considered insulin resistant. Compared to other established indices studied (HOMA-IR, HOMA2-IR, HOMA2-IR, QUICKI, McAi and TG/HDL), authors found that the TyG index was the most robust index for determining IR in the Qatari population and could be useful in Middle Eastern populations for IR screening.

Duan et al. investigated a somewhat neglected complication/co-morbidity of T2D, *viz.* depression. Depression is highly prevalent among T2D patients and also increases mortality even further in these patients already at high risk for cardiovascular death (18). The causal relationship between T2D and depression remains elusive; do the patients get depression because of their chronic diabetes and the burdens imposed thereof, or *vice versa* (*i.e.*, what is the chicken and what is the egg)?. Depression also carries diabetes-specific problems: reduced energy for physical activity leading to weight gain and hyperglycemia, impaired self-care and compliance to treatment, emotional eating behavior, *et cetera*. The current study aimed to identify key factors influencing depression, such as the relationship between the Cumulative Illness Rating Scale (CIRS) score and depression, and to evaluate the predictive value of a model incorporating sex, body mass index (BMI), low-density lipoprotein cholesterol (LDL-C), and CIRS score. The authors found that female sex, lower BMI, lower LDL-C and higher CIRS score were independently associated with depression in T2D patients and propose a more comprehensive prediction model that could help identify T2D patients with at risk for depression.
